# Seroepidemiology of *Paracoccidioides brasiliensis* infection in horses from Rio Grande do Sul, Brazil

**DOI:** 10.1590/S1517-838246246220140559

**Published:** 2015-06-01

**Authors:** Ana Paula Neuschrank Albano, Gabriel Baracy Klafke, Tchana Martinez Brandolt, Vanusa Pousada Da Hora, Carlos Eduardo Wayne Nogueira, Melissa Orzechowski Xavier, Mário Carlos Araújo Meireles

**Affiliations:** 1 Universidade Federal de Pelotas, Departamento de Veterinária Preventiva, Faculdade de Veterinária, Universidade Federal de Pelotas, Pelotas, RS, Brasil, Departamento de Veterinária Preventiva, Faculdade de Veterinária, Universidade Federal de Pelotas, Pelotas, RS, Brazil.; 2 Universidade Federal do Rio Grande, Laboratório de Micologia, Faculdade de Medicina, Universidade Federal do Rio Grande, Rio Grande, RS, Brasil, Laboratório de Micologia, Faculdade de Medicina, Universidade Federal do Rio Grande, Rio Grande, RS, Brazil.

**Keywords:** ELISA, gp-43, protein G-peroxidase, paracoccidioidomycosis

## Abstract

*Paracoccidioides brasiliensis* is the etiological agent of the major systemic mycosis in Brazil, called paracoccidioidomycosis. Although the Rio Grande do Sul is considered an endemic area of the disease, there are few studies on the ecology of *P. brasiliensis* in the state. Therefore, this study aimed to evaluate the infection of *P. brasiliensis* in horses from the mesoregion of Southwest Riograndense, using these animals as sentinels. Serological techniques, such as double immunodiffusion in agar gel (AGID) and indirect ELISA, were performed to detect the anti-gp43 *P. brasiliensis* antibody in horses from five different farms in the region of Bagé, RS, Brazil. Serology was performed in 200 Pure Blood English horses up to two years of age that were born and raised exclusively at the farms. Of these horses, 12% had anti-gp43 antibodies according to the ELISA results, with rates ranging from 0 to 30% according to the farm of origin (p < 0.001). Based on the immunodiffusion results, all equine serum samples were negative. These results indicate the presence of the fungus *P. brasiliensis* in the middle region of the southwestern state of Rio Grande do Sul, Brazil.

## Introduction

The thermally dimorphic fungus *P. brasiliensis* is the etiological agent of the major systemic mycosis in Brazil, called paracoccidioidomycosis. This microorganism has a limited geographical distribution and is found in all regions from northern Argentina to southern Mexico, with the exception of Guyana, French Guiana, Suriname, Chile and Nicaragua (Brummer *et al.*, 1993; Bártholo *et al.*, 2000; Marques, 2003).

The infection of susceptible individuals occurs by inhalation of fungal propagules in their filamentous phase that are found in the environment. Although there are other hypotheses, wet soil is considered the main habitat of *P. brasiliensis* (Conti-Diaz, 2007). However, studies on the ecological niche of *P. brasiliensis* are still inconclusive, and the traceability of the fungus in the environment is very difficult due to the absence of outbreaks and poor repeatability observed in fungal isolation from the environment, as well as to variable period between infection and illness (latency), which impairs the associations among the source of infection (Bagagli and Bosco, 2008).

Therefore, studies using animals as sentinels have been important to prove the presence of the fungus in certain regions. Although paracoccidioidomycosis is endemic in Rio Grande do Sul (RS) for decades, we recently described for the first time the fungus presence in two mesoregions of the state (Albano *et al.*, 2014). Thus, this study aimed to evaluate the presence of *P. brasiliensis* in another mesoregion, the Southwest Riograndense mesoregion, by seroepidemiology of horses from different stables. This region is characterized by the pampas biome, which is completely different from the rest of the country; this biome is found only in the southern half of the state of Rio Grande do Sul (Brazil), Uruguay and Argentina.

## Material and Method

**Figure 1:**
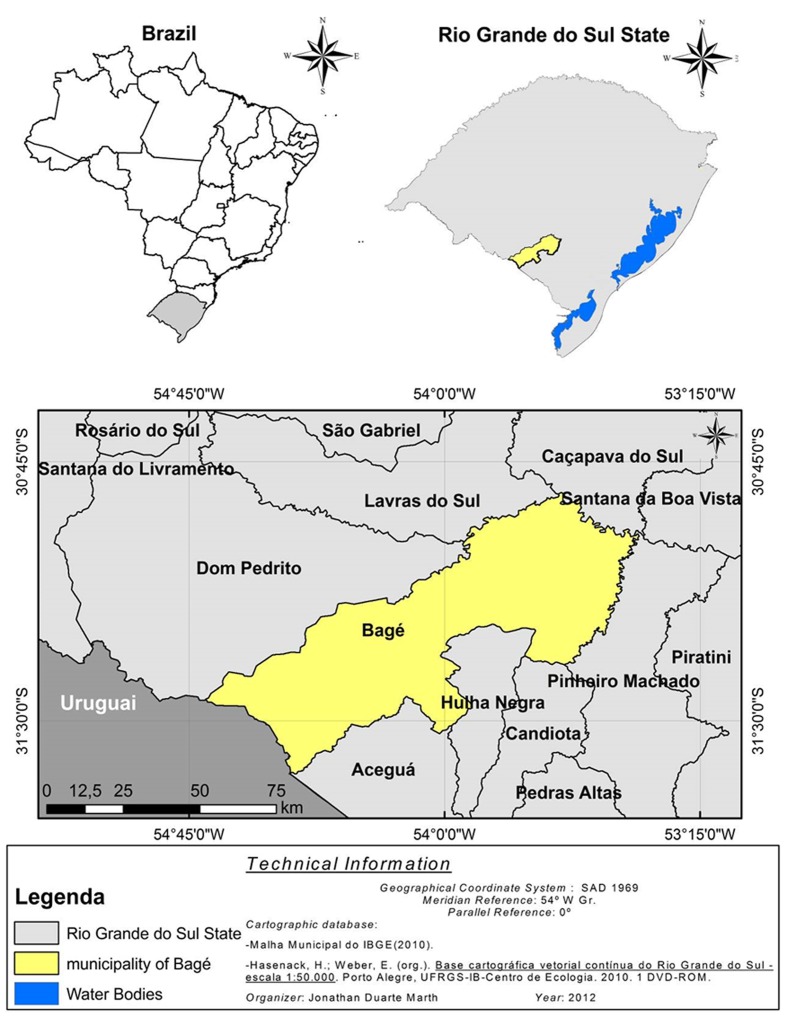
Map showing the city of Bage, Rio Grande do Sul, Brazil.

The study was conducted with animals from the city of Bage, which is located in the Gaucho Pampas, the microregion of Southern Campaign that belongs to the middle region of the Southwest Riograndense region, Rio Grande do Sul, Brazil (Figure 1). It has a mean altitude of 212 m, a climate that can be characterized as either temperate or subtropical, tepid summers (*i.e.*, high temperatures during the day and moderate temperatures at night), and fairly cold winters with frost. The average annual temperature is 18 °C, and the monthly mean temperatures range from 12 °C in winter to 24 °C in summer. Rainfall is usually evenly distributed throughout the year, and the average annual volume is 1,472 mm, with monthly averages ranging from 104 mm to 142 mm (FEE, 2013).

Two hundred thoroughbred horses (Pure Blood English), up to two years old and born and raised exclusively at five different farms in the city of Bage, were included in the study. The animals were randomized, and those who had access to other locations before (*e.g.*, transportation, participation in events, auctions) were excluded.

From each animal, data about the age, gender, and farms of origin, including geographical location, were collected. All animals were subjected to a single blood sample collection for serological analysis by puncture of the jugular vein. After separation of the serum, samples were stored at −20 °C until two serological tests were performed: the double radial immunodiffusion in agar gel (AGID) test and an enzyme linked immunosorbent assay (ELISA) using the fungal antigen gp43, a glycoprotein of 43 kDa, to detect specific IgG anti-*P. brasiliensis*.

The exoantigen was obtained as described by Camargo *et al.* (1988) using the *P. brasiliensis* isolate B-339. The gp43 antigen was purified from the *P. brasiliensis* exoantigen by affinity chromatography according to Puccia and Travassos (1991), and the protein concentration was determined via the Bradford method using BSA as a standard.

For the ELISA test, polystyrene microplates of 96 wells (Corning Costar Corporation, Corning, NY, USA) were sensitized with gp43 (260 ng/well) in 100 μL of carbonate/bicarbonate buffer (pH 9.6) and incubated at 4 °C for 18 h. The plates were then washed with phosphate buffered saline with 0.5% Tween 20 (PBS-T) and blocked with PBS-1% milk (skimmed milk powder diluted in PBS) for 60 min at 37 °C. Then, the plate was washed five times with PBS-T and incubated for 60 min at 37 °C with the test serum diluted 1:100 in PBS. After washing five times with PBS-T, 100 μL of conjugate (protein G -peroxidase conjugate, Sigma ®, 1:10000) was added to each well, followed by incubation for 60 min at 37 °C. Following the final wash performed by ten times with PBS-T, 100 μL of substrate/chromogen (4 mg of OPD dissolved in 10 mL citrate buffer) was added. After incubation for ten min at 37 °C, the reaction was blocked by the addition of 100 μL of 1N sulfuric acid, and absorbance was determined in a microplate reader (TECAN Spectra classic) using a 450 nm filter. All samples were tested in triplicate. As a positive control, a commercially available serum (*Paracoccidioides* ID positive control, Immuno Mycologics, Inc., IMMY®), was used, and the serum negative control corresponded to a "pool" of negative sera from previously tested horses, which showed absorbance at approximately 0.07 to 0.10 (CUT, 2009), all equally diluted 1:100 in PBS. Samples with absorbance greater than twice the negative control were considered positive.

The immunodiffusion test was conducted with the gp43 antigen on the central orifice and the samples to be tested in the side holes. The serum positive control (*Paracoccidioides* ID positive control - Immuno Mycologics, Inc.) was used in the reaction in the top and bottom holes for reading and for the final interpretation of the test, as described by Camargo *et al.* (1988).

The study was conducted according to the standards of animal welfare and was approved by the Ethics and Animal Experimentation of the Federal University of Pelotas (EAEC n°7123). The results were analyzed by chi-square test for categorical variables with SPSS® 20.0, and p-values less than 0.05 were considered statistically significant.

## Results

Of the 200 horses studied, 102 were females and 98 males. All serum samples were negative in the AGID test for the detection of anti-gp43 of *P. brasiliensis*. Nevertheless, the ELISA showed seropositivity in 24 individuals, representing 12% of the population. Of these, eleven animals were females, and thirteen were males (p = 0.589).

When the seropositivity of animals according to the farm of origin was analyzed, a significant difference (p < 0.001) was found, with two properties showing 30% positivity and one farm without any positive animal (Table 1).

**Table 1:** Results of the ELISA test for anti-gp43 of P. brasiliensis in horses according to their place of origin.

Place	Total animals studied	Seropositive n (%)
1	30	9 (30)^A^
2	103	3 (2.9)^B^
3	30	9 (30)^A^
4	12	0 (0) ^B^
5	25	3 (12)^AB^
Total	200	24 (12)

Uppercase letters correspond to the results of statistical analysis using chi-square test, where the same letters mean p > 0.05 and different letters means p < 0.05.

## Discussion

This study evaluated the seropositivity of horses for the fungus *P. brasiliensis* in the mesoregion of Southwest Riograndense of Rio Grande do Sul, Brazil.

In agreement with several other studies, none of the animals were seropositive according to the results of the double radial immunodiffusion technique (AGID) (Ono *et al.*, 2001; Corte *et al.*, 2009; Canteros *et al.*, 2010). However, the ELISA assay detected seropositivity in 12% of the horses studied. Corte *et al.* (2007) also detected low or no seropositivity in black howler monkeys (*Alouatta caraya*) and capuchin monkeys (*Cebus spp.*) by AGID, although IgG anti-gp43 was detected by ELISA in 60% and 44.1% of these animals, respectively.

This discrepancy in results found between techniques is associated with their sensitivity (Ono *et al.*, 2001). The AGID technique is less sensitive, though it is more accessible because it is less expensive and easier to use. The AGID is based on a precipitation reaction that shows positive results only in serum samples with a large concentration of circulating antibodies, which is usually found in individuals with an active form of the disease of interest. For this reason, the AGID technique can be used as a diagnostic tool for paracoccidioidomycosis and is considered the gold standard for this disease. However, it is not able to detect exposure to the fungus (Palmeiro *et al.*, 2005; Ramos e Silva and Saraiva, 2008). Conversely, to detect this asymptomatic infection, the ELISA technique is more effective, allowing for identification subjects even with low concentrations of circulating antibodies, making it ideal for seroepidemiologic studies of fungal exposure.

The seropositivity rate of approximately 12% of the animals used as sentinels for *P. brasiliensis* in this study is similar to that described in other states of Brazil, such as Minas Gerais, Mato Grosso do Sul and Paraná, where authors have described a exchange seropositivity of 17.5% in a study with dairy cattle(Silveira *et al.*, 2008), 30% in a study with horses (Corte *et al.*, 2009) and 37% in a study with sheep(Oliveira *et al.*, 2012). However, in the state of São Paulo, which is considered a hyperendemic area of PCM, previous authors have described even higher reactivity rates (*i.e.*, 63.8% and 40.8%) of animals to the antigen of *P. brasiliensis* using an intradermal test (Costa *et al.*, 1995); another study with 126 urban dogs in the Amazon described 54.8% seropositivity (Corte *et al.*, 2009). In Rio Grande do Sul, few similar studies have been conducted to allow for a comparison of the results obtained in different mesoregions of the state. According to the results of Albano *et al.* (2014), which evaluated 128 wild animals as sentinels for the presence of *P. brasiliensis*, a positivity of 20% was found, of which 76.92% were from the Southeast mesoregion and 23.07% were from the Metropolitan mesoregion city of Porto Alegre. Another serological study in Uruguay, a country with similar climatic conditions to those found in the state of RS, showed a seropositivity rate of 23% among the horses tested, which is close to the values described in our results (Conti-Diaz *et al.*, 1972).

As described in other serological studies that used domestic dogs and primates, the seropositivity did not differ in relation to the gender of the animals studied, suggesting that both are equally infected by *P. brasiliensis* infective propagules in the environment, most likely because the animals must remain in direct contact with the ground, the natural habitat of the fungus, since birth (Ono *et al.*, 2001; Silveira *et al.*, 2006; Corte *et al.*, 2007; Corte *et al.*, 2009).

The significant difference found in the seropositivity in horses in relation to farms of origin may indicate that specific environmental factors of each property could influence the fungus survival in the soil. Though none of the properties uses pesticides and they all have similar types of vegetation (pasture, improved native pasture), the soil characteristics, such as pH, humidity and concentration of organic matter and aluminum ions, should be evaluated to confirm the presence/absence of *P. brasiliensis* (Terçarioli *et al.*, 2007). In fact, according to Terçarioli *et al.* (2007), *P. brasiliensis* grows in clay or sandy soil in high humidity and is inhibited in soils with high concentrations of exchangeable aluminum (Al + H). In addition, the farms' proximity to water courses should also be evaluated due to the hypothesis supported by Conti-Diaz (2007) that water is another ecological niche for *P. brasiliensis*. The water gives it a highly efficient ecological strategy, characterized by the use of heterothermic animals living in natural freshwater sources during a provisory period; therefore, the soil could be only the transient habitat of the fungus (Conti-Diaz, 2007; Restrepo *et al.*, 2001). Thus, more studies are needed to elucidate the eco-epidemiology of *P. brasiliensis* in southern Brazil.

## Conclusion

Specific IgG antibodies against *P. brasiliensis* were detected in the serological study of horses from the region of Bage, showing that the fungus is found in the mesoregion of Southwest Riograndense, Brazil.
